# Double oscillating diffusion encoding and sensitivity to microscopic anisotropy

**DOI:** 10.1002/mrm.26393

**Published:** 2016-08-31

**Authors:** Andrada Ianuş, Noam Shemesh, Daniel C. Alexander, Ivana Drobnjak

**Affiliations:** ^1^ Centre for Medical Image Computing, University College London London UK; ^2^ Champalimaud Neuroscience Programme, Champalimaud Centre for the Unknown Lisbon Portugal

**Keywords:** double diffusion encoding, multiple diffusion encoding, microscopic anisotropy, microstructure, double‐PFG, oscillating gradient spin echo, OGSE, MRI

## Abstract

**Purpose:**

To introduce a novel diffusion pulse sequence, namely double oscillating diffusion encoding (DODE), and to investigate whether it adds sensitivity to microscopic diffusion anisotropy (µA) compared to the well‐established double diffusion encoding (DDE) methodology.

**Methods:**

We simulate measurements from DODE and DDE sequences for different types of microstructures exhibiting restricted diffusion. First, we compare the effect of varying pulse sequence parameters on the DODE and DDE signal. Then, we analyse the sensitivity of the two sequences to the microstructural parameters (pore diameter and length) which determine µA. Finally, we investigate specificity of measurements to particular substrate configurations.

**Results:**

Simulations show that DODE sequences exhibit similar signal dependence on the relative angle between the two gradients as DDE sequences, however, the effect of varying the mixing time is less pronounced. The sensitivity analysis shows that in substrates with elongated pores and various orientations, DODE sequences increase the sensitivity to pore diameter, while DDE sequences are more sensitive to pore length. Moreover, DDE and DODE sequence parameters can be tailored to enhance/suppress the signal from a particular range of substrates.

**Conclusions:**

A combination of DODE and DDE sequences maximize sensitivity to µA, compared to using just the DDE method. Magn Reson Med 78:550–564, 2017. © 2016 The Authors Magnetic Resonance in Medicine published by Wiley Periodicals, Inc. on behalf of International Society for Magnetic Resonance in Medicine. This is an open access article under the terms of the Creative Commons Attribution License, which permits use, distribution and reproduction in any medium, provided the original work is properly cited.

## INTRODUCTION

Diffusion Magnetic Resonance Imaging has become one of the most important probes of tissue microstructure with many applications in biomedical imaging 1, 2. Microscopic diffusion anisotropy (µA) 3 in particular, is a measure which reflects local anisotropy at the cellular level and depends on the compartment size and shape, providing valuable information on white and grey matter structure 5, 6, 17, different tumor types 6 as well as understanding structural changes in diseases such as stroke 7.

The typical approach to estimate microscopic anisotropy uses Double Diffusion Encoding (DDE) 3, 8, 9 sequences, which concatenate two independent gradient pairs separated by a mixing time, measuring the correlation of water displacement in different directions. In the long mixing time regime, DDE sequences that vary the relative angle between the two gradient wave vectors are sensitive to µA 9, even in heterogeneous substrates 10. The amplitude of the signal modulation in angular DDE experiments 9, 11 reports on µA, reflecting the eccentricity of the pores, which can be independent of orientation dispersion when rotationally invariant DDE protocols are considered 12, 13, 14. Metrics of µA have been quantified in cells 11, 15, tissues 16, 17, 18, ex vivo and in vivo animal brains 19, and even in an animal model of stroke where intracellular µA was observed for metabolites 7; as well, DDE‐derived µA was quantified in vivo on clinical scanners 5, 20, 21, and have provided intriguing contrasts especially in the gray matter, a typically highly disordered neural tissue. Microscopic anisotropy can also be estimated using other diffusion acquisitions which vary the gradient direction in one measurement 6, 22, 23, 24, as well as single diffusion encoding with appropriate modeling constraints, e.g., identical micro‐domains 25, yet the relative accuracy, vis‐á‐vis estimates from DDE measurements, remains to be determined 26. In biological tissue, µA is determined by microstructural properties such as pore size and eccentricity. Therefore, increasing the sensitivity to these features can improve the estimates of µA.

**Figure 1:**
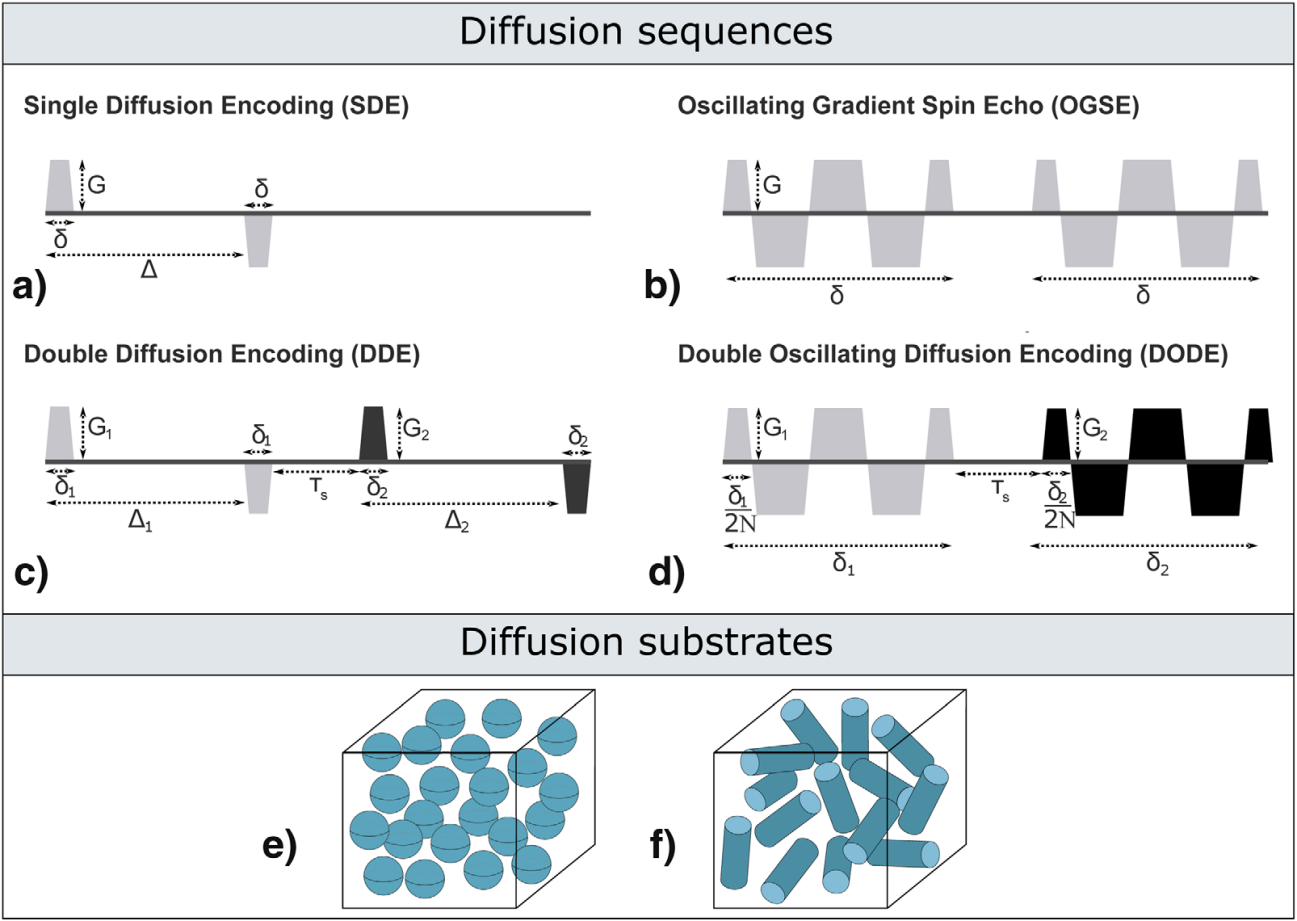
**a–d**: Schematic representation of diffusion gradient waveforms. Notice that in b and d, the oscillating gradients have cosine‐like waveforms. **e–f**: Schematic representation of diffusion substrates featuring microscopically isotropic and anisotropic pores.

Recent work has shown that replacing the pulsed gradients with oscillating gradients in single diffusion encoding (SDE) experiments can be beneficial for imaging pore sizes 27, 28, 29. SDE sequences 30 have been used for studying compartment size in various approaches: for example, q‐space imaging 31, 32 can provide insights into the characteristic compartment size and orientation by reconstructing the diffusion propagator 31, 33. Other techniques use geometric models of restriction to estimate pore size 34. This led to the development of biophysical models for the diffusion signal which can facilitate the estimation of various microstructural tissue features, such as axon diameter distribution 35 or index (volume weighted average) 36, 37, 38, 39 in parallel or dispersed fibres 40, 41 as well as cell size in tumors 42, 43. Oscillating gradient spin echo (OGSE) sequences, illustrated in Figure 1b, a whole range of effective diffusion times can be probed, including short values which cannot be practically achieved using SDE sequences, providing additional information about the substrate 29, 44, 45, 46, 47, 48, 49. Different oscillating gradient waveforms, such as non‐uniform‐OGSE 50, 51, 52 or numerically optimized waveforms, have been also proposed to further increase the sensitivity to small sizes. Circularly polarized OGSE sequences, which provide encoding in a plane rather than a single direction, can further increase the diffusion weighting and improve contrast 53. A recent study explicitly compared in simulation the sensitivity of SDE and OGSE sequences to axon diameter, showing that OGSE has major advantages in the presence of fiber dispersion or slightly offset gradient directions 28.

In this study, we introduce the double oscillating diffusion encoding (DODE) sequence. We demonstrate, in simulation, that DODE sequences provide increased sensitivity to microscopic anisotropy compared to DDE, and we explore the specificity of the signal amplitude modulation for a wide range of substrates and sequence parameters. DODE sequences are achieved by replacing the two diffusion encoding gradient pairs in DDE with two independent oscillating gradient waveforms which can accommodate different orientations. Then, we investigate the dependence of DODE signals on sequence parameters and compare the sensitivity of DDE and DODE to microstructural features. Specifically, in heterogeneous substrates featuring randomly oriented elongated pores, modeled as finite cylinders, we analyse the sensitivity to pore dimensions which directly influence µA, i.e., diameter and length. Furthermore, we study the ability of DDE and DODE sequences to quantify µA at various length scales.

## METHODS

### DODE and DDE Sequences

DODE sequences consist of two oscillating gradient waveforms whose orientations are independent replacing the two pairs of pulsed gradients in the DDE sequence. Figure 1c,d schematically illustrates the standard DDE as well as the newly introduced DODE sequences and their parameters. In this work, we restrict the two gradient pulses in both DDE and DODE to have the same parameters except for gradient orientation, which is the parameter subspace used for estimating pore size and µA in conventional DDE studies. Thus, DDE sequences are described by gradient amplitude $G=G_{1}=G_{2}$, pulse duration $\delta =\delta_{1}=\delta_{2}$, diffusion time $\Delta =\Delta_{1}=\Delta_{2}$, separation time *τ_s_* (time interval between the two pairs of gradients) and the relative angle between gradient orientations *ψ*. (Note: the mixing time usually defined for a DDE sequence corresponds to *τ_s_* + Δ). The parameters of the DODE sequence are the gradient amplitude $G=G_{1}=G_{2}$, gradient duration $\delta =\delta_{1}=\delta_{2}$, number of half oscillation periods $N=N_{1}=N_{2}$, separation time *τ_s_* (time interval between the two gradient waveform) and the relative angle between gradient orientations *ψ*. The oscillating gradients in the DODE sequence considered here have cosine‐like waveforms, which yield a single main peak in the power modulation spectrum 54 at the oscillation frequency of the gradient. For all sequences we assume a finite gradient slew rate which can be achieved on modern preclinical scanners, i.e., SR = 1000 T/m/s, unless specified otherwise.

### Diffusion Substrates and Simulation Framework

Substrates with a range of different microscopic anisotropy values, from highly isotropic ones (spheres, Fig. 1e) to highly anisotropic ones (cylinders, Fig. 1d) are considered. We characterize µA through pore dimensions, namely diameter *d* and length *L*. A substrate of finite cylinders provides a representation of diffusion inside cells of various elongations, such as in tumor tissue 6. In all simulations, we consider only intracellular signal, unless otherwise specified, and set the intrinsic diffusivity to $D=2\cdot 10^{-9}$m^2^/s, a value similar to the principal eigenvalue of the diffusion tensor measured at short diffusion time in the human brain in vivo 55. To reduce the parameter space, we investigate macroscopically isotropic substrates, and limit the acquisition protocols to a single orientation of the first gradient pulse. However, orientationally invariant DODE protocols can be constructed in a similar way to orientationally invariant DDE protocols, as in 5, 12, 14.

The simulations in this paper have been performed using MISST (Microstructure Imaging Sequence Simulation Toolbox—http://www.nitrc.org/projects/misst). The diffusion signal is calculated using the matrix formalism from 56 and the implementation for gradients with varying orientation detailed in 27, 57.

### Study 1: Qualitative Comparison of DODE and DDE

The first study compares the dependence of DODE and DDE signal on sequence parameters in substrates featuring microscopic diffusion anisotropy and tests whether DODE gives similar trends as DDE when the angle between the two gradients, *ψ*, is varied. Specifically, we analyze the amplitude of the signal modulation as a function of *ψ* for sequences with different separation times and oscillation frequencies.

#### Effect of Varying Separation Time

In the first simulation, we compare the effect of increasing separation time *τ_s_* on the amplitude of the signal modulation for DODE and DDE sequences in a substrate featuring randomly oriented anisotropic pores with diameter *d* = 4 µm and length *L* = 12 µm. To study a similar diffusion regime, we fix the *b*‐values for both sequences to b={3000,5000} s/mm^2^ and modify the gradient strength accordingly. For DODE, we consider the following parameters: gradient duration *δ* = 50 ms, separation time $\tau_{s}=\{0,10,20,30,50\}$ ms, and number of half periods N={2,4} with corresponding gradient amplitude G={90,180} mT/m for *b* = 3000 s/mm^2^ and G={116,232} mT/m for *b* = 5000 s/mm^2^. The DDE sequence parameters are: gradient amplitude G={111,143} mT/m, pulse duration δ=6.25 ms (which correspond to the duration of each half period for the DODE sequence with *N* = 4), diffusion time Δ = 50 ms and separation time $\tau_{s}=\{0,10,20,30,50\}$ ms. To better understand the effects of separation time on the DODE and DDE signal, we further analyse the power spectra of the two sequences, when $\tau_{s}=0$ and the gradient directions are either parallel or antiparallel.

#### Effect of Varying Oscillation Frequency

In the second simulation, we investigate the effect of varying the number of oscillation half periods *N* on the amplitude of the DODE signal modulation, in substrates with different degrees of diffusion anisotropy. We consider randomly oriented finite cylinders with diameter *d* = 4 µm and two different lengths L={12,8} µm as well as spherical pores with *d* = 4 µm. First, to understand the effect of varying the gradient frequency, rather than decreasing the amount of diffusion weighting, we analyze sequences that have the same *b* = 5000 s/mm^2^. We evaluate the dependence of the DODE signal on *ψ* for various number of oscillation periods N={2,4,8,12,18}. The rest of the sequence parameters used in the simulation are: *δ* = 50 ms, $\tau_{s}=20$ ms, and gradient strength G={89,179,361,556,1031} mT/m which is adjusted to yield the same *b*‐value. As in practice there is a physical constraint on the maximum gradient strength, we also investigate the case when DODE sequences with different *N* have the same gradient strength *G* = 300 mT/m.

### Study 2: Sensitivity of DODE and DDE Signal to Pore Size and Length

The second study compares the sensitivity of DODE and DDE sequences to pore diameter and length, the microstructural features that determine µA. This provides insight into the sequence parameters that provide optimal contrast to different substrates.

As measurements with parallel and perpendicular gradients are of interest in microscopically anisotropic pores, we describe the total sensitivity as the sum of sensitivities for these two measurements. For one measurement, we define the sensitivity as the absolute value of the partial derivative with respect to the parameter of interest. Thus, the total sensitivities are:

(1) $$S_{d}=\left|\partial_{d}\left(S_{∥}(d,L)\right)\right|+\left|\partial_{d}\left(S_{⊥}(d,L)\right)\right|,$$

with respect to pore diameter and

(2) $$S_{L}=\left|\partial_{L}\left(S_{∥}(d,L)\right)\right|+\left|\partial_{L}\left(S_{⊥}(d,L)\right)\right|,$$

with respect to pore length, where *d* is the pore diameter, *L* is the pore length, $S_{∥}$ is the diffusion signal measured from sequences with parallel gradients, and $S_{⊥}$ is the signal obtained from measurements with perpendicular gradients.

#### Sensitivity for a Wide Range of Substrates

The first simulation compares the sensitivity of several DODE and DDE sequences in substrates with a large variety of parameters. We consider randomly oriented infinite cylinders with diameter *d* between 1 and 12 µm as well as randomly oriented finite cylinders with d={4,6} µm and a range of lengths *L* between 4 and 40 µm. We analyze DODE sequences with various numbers of oscillations, DDE sequences with finite gradient duration as well as ideal DDE sequences with short gradient duration. As in practice the gradient strength is a physical constraint, in this simulation, we fix the gradient strength of the DODE sequences to *G* = 300 mT/m, corresponding to the Connectome scanner 58. The rest of the DODE parameters are: *δ* = 50 ms, $\tau_{s}=20$ ms, and N={1,2,4,12,18}. (*Note: DODE sequences with N = 1 are equivalent to DDE sequences with δ* = Δ). For the DDE sequences, we look at 2 different scenarios, and in all cases Δ = 50 ms and $\tau_{s}=20$ ms:

1. DDE sequences have the same gradient amplitude *G* = 300 mT/m and various pulse durations equal to half the oscillation period of the DODE sequences: δ={25,12.5,6.3,2.1,1.4} ms.
2. The gradient amplitude of idealized DDE sequences with *δ* = 1 ms and infinite slew rate is adjusted to get matching *b*‐values with the DODE sequences for each *N*. The resulting gradient strengths are G={4.34,2.17,1.09,0.36,0.24} T/m. The *b*‐values for DODE sequences are computed using the expressions derived in 59 for OGSE sequences. Although the gradient strength and slew rate for matching the *b*‐values becomes unrealistically high, it provides a useful theoretical comparison.

#### Sensitivity for a Wide Range of Sequence Parameters

The second simulation investigates the sensitivity of DODE and DDE sequences over a wide range of practical sequence parameters in several substrates, which consist of randomly oriented finite cylinders with diameter d={4,6} µm and eccentricities of L/d={1,2,4,8}. We make the two sequences equally practical by ensuring the same maximum gradient strength and maximum duration for both DODE and DDE sequences. The range of parameters for DODE sequences are: G=[0,400] mT/m, $\delta_{\text{DODE}}=[0,50]$ ms and N={1,2,4,6,8,10}. For the DDE sequences the range of gradient strengths is the same G=[0,400] mT/m and we consider five different diffusion times Δ={25,30,35,40,45} ms. For each Δ we have a different range of gradient durations *δ* in order to limit the total duration of each gradient pair ( δ+Δ) at 50 ms. In order to reduce the dimensionality of the problem, the separation time is fixed to 20 ms for all sequences. Additionally, we investigate the more realistic scenario where sequences with a longer duration are penalized due to T2 decay. Thus we analyze the effect of T2 relaxation with a constant of 70 ms, which is in the range of values for gray matter at 3T.

### Study 3: Specificity to Microscopic Anisotropy

As the difference between DODE/DDE measurements with parallel and perpendicular gradients is a signature of microscopic anisotropy, the last simulation investigates how different sequence parameters influence this contrast in a large variety of substrates. This facilitates the design of experiments which improve the specificity to the microstructural features of interest. Thus, we analyze the signal difference between the two sets of measurements for DODE and DDE sequences with different varying parameters in a wide range of substrates with pore diameters 0.5 µm <d<10 µm and eccentricities 1<L/d<10. For DODE sequences we vary independently *G*, *N*, and *δ*, while for DDE we vary *G*, *δ*, and Δ. For both sequences, the time interval between the two gradients has a constant value of 20 ms. We also analyze the effect of noise and label the regions where the difference is larger than the standard deviation of the noise for different levels of SNR={20,50,100,1000}. This highlights which substrates can be distinguished from isotropic pores, given the diffusion sequence and SNR level.

## RESULTS

### Study 1: Qualitative Comparison of DODE and DDE

The first set of simulations investigates the diffusion signal of DODE sequences for various parameters and compares the results with the well‐established angular signal dependence of DDE sequences in the presence of microscopic anisotropy.

#### Effect of Varying Separation Time

**Figure 2:**
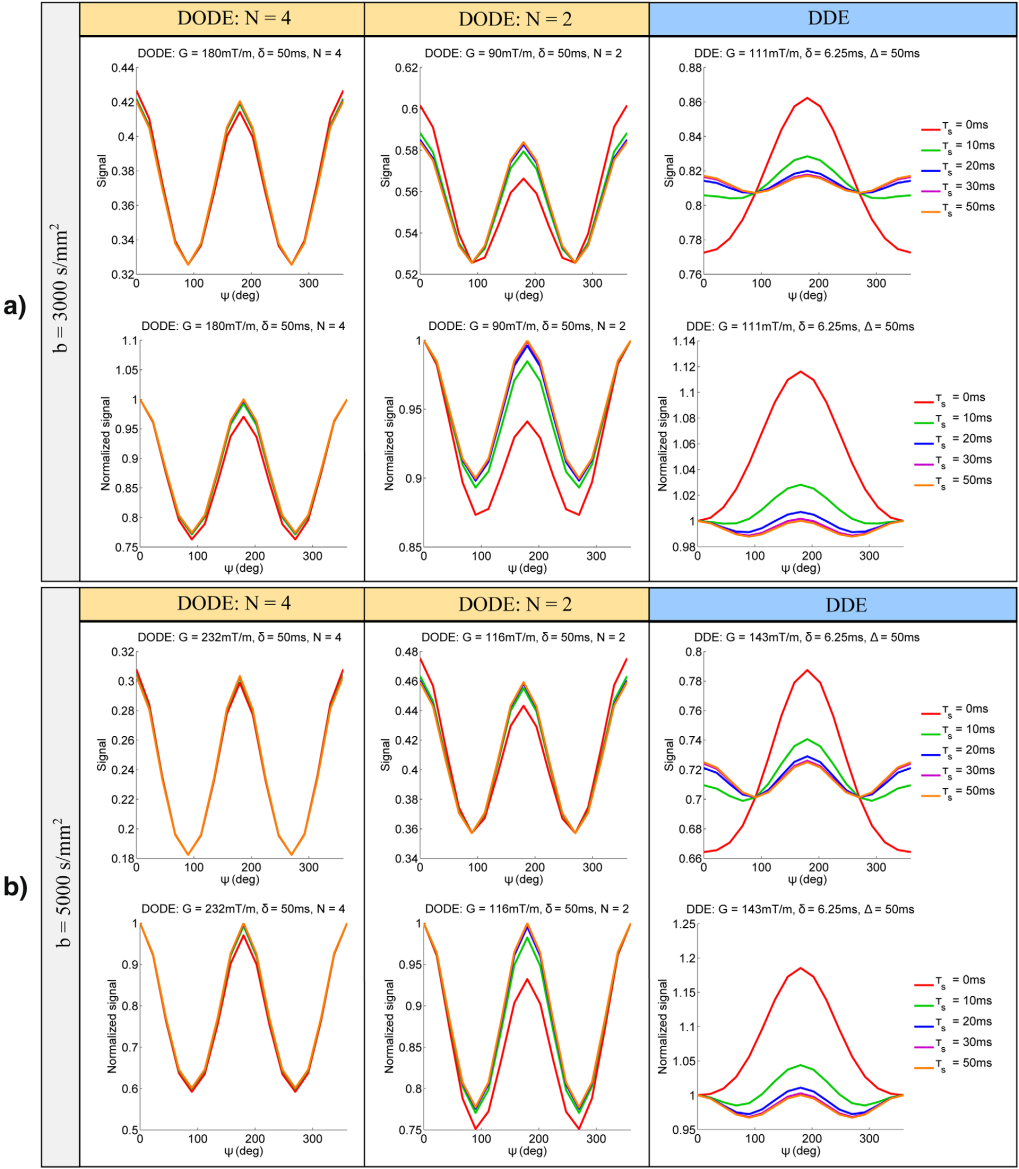
Signal and normalized signal as a function of the angle between gradients for DODE and DDE sequences with various mixing times and **a**: *b* = 3000 s/mm^2^ and **b**: *b* = 5000 s/mm^2^. The diffusion substrate consists of randomly oriented finite cylinders with diameter *d* = 4 µm and length *L* = 12 µm.

Figure 2 illustrates the dependence of the DODE and DDE signal on the angle between the two gradients, *ψ*, for various separation times, for a substrate of randomly oriented finite cylinders with diameter *d* = 4 µm and length *L* = 12 µm. The signal itself as well as the normalized signal with respect to the measurements with parallel gradients are plotted in Figure 2a for sequences with *b* = 3000 s/mm^2^ and in Figure 2b for sequences with *b* = 5000 s/mm^2^. Both the DODE and DDE signals exhibit an angular dependence on *ψ*, however, the influence of the separation time differs for the two sequences. For zero separation time (*τ_s_* = 0 ms), DDE sequences exhibit the well‐described 9, 16, 60 bell‐shaped signal dependence, with the largest signal difference between measurements with parallel and antiparallel gradients, which is an indication of restricted diffusion 16, 17, 18. As the separation time increases, the signal dependence resembles the expected cos(2*ψ*) function 9, 10, 61, with the largest signal difference corresponding to measurements with parallel and perpendicular gradients. This amplitude modulation is a signature of µA 9.

**Figure 3:**
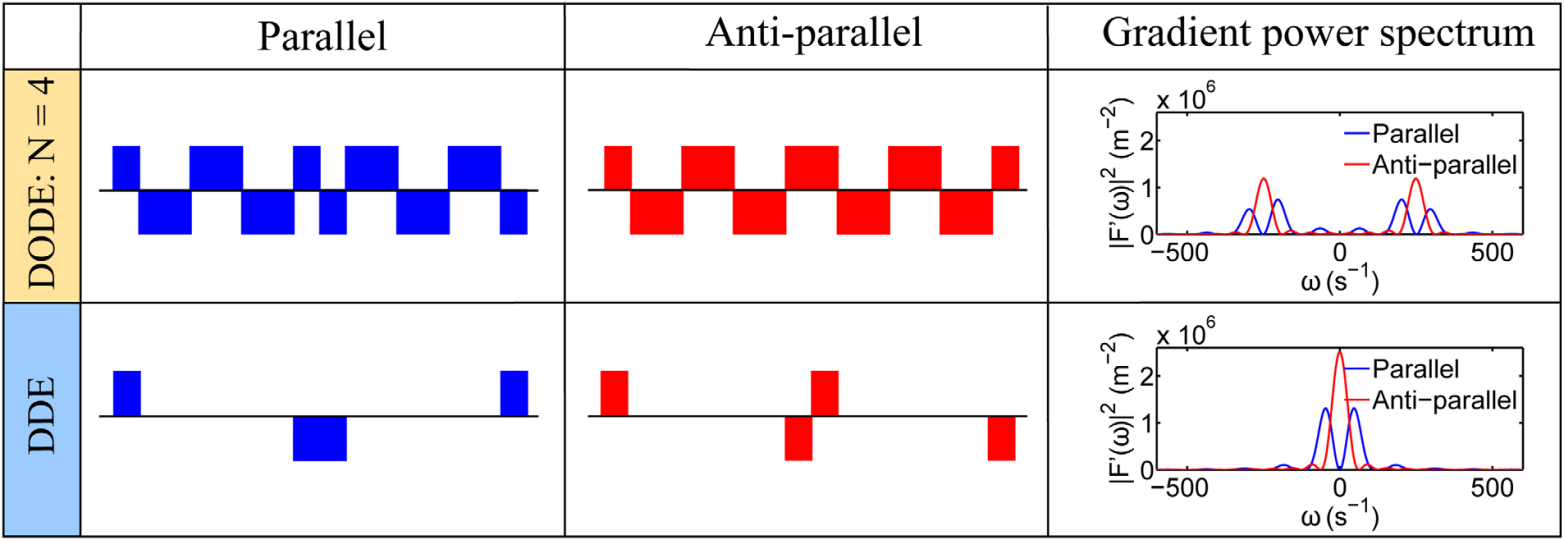
Power spectrum of DODE (*N* = 4) and DDE sequences with parallel and antiparallel gradient orientation.

For DODE sequences, the angular dependence has a similar trend, however, the influence of separation time becomes less pronounced as the frequency is increased, which is illustrated in Figure 2 for DODE with N={2,4}. For sequences with *N* = 4, the signal difference between measurements with parallel and antiparallel gradients becomes close to zero even for short time intervals between the two gradient waveforms, which, for the standard DDE sequences, is characteristic of the long mixing time regime. Frequency domain considerations can be used to better explain the observed effects of separation time on the signal for DDE and DODE sequences. The power spectrum, defined as the Fourier Transform of the gradient integral, indicates which part of the diffusion spectrum is sampled by the gradient. For DODE and DDE sequences with zero separation time ( $\tau_{s}=0$), changing the orientation of the second gradient from parallel ( $\psi =180^{{}^{\circ}}$) to antiparallel ( $\psi =180^{{}^{\circ}}$) causes a split in the main peak of the power spectrum, as illustrated in Figure 3 for DODE with *N* = 4 and DDE sequences. For DDE sequences, this peak is around zero‐frequency, and the diffusivity values sampled in the two cases are very different, which causes a large signal difference between measurements with parallel and antiparallel gradients, as seen in Figure 2. As the frequency increases, the diffusivity values sampled by parallel and antiparallel DODE sequences become more similar, yielding a small signal difference between the two measurements, as seen in Figure 2 for DODE with *N* = 4.

#### Effect of Varying Oscillation Frequency

**Figure 4:**
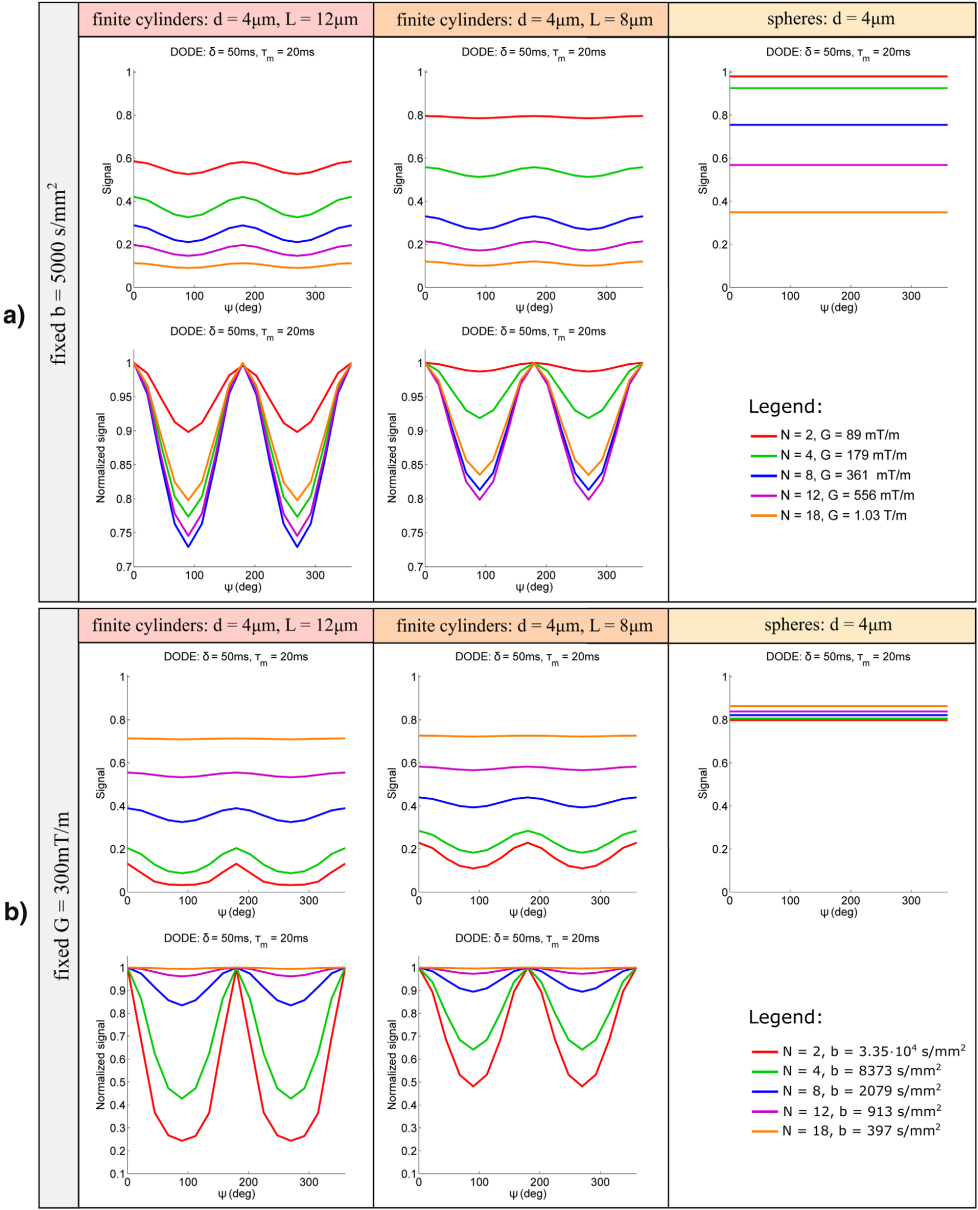
Signal and normalized signal as a function of the angle between gradients for pores with different eccentricities and DODE sequences with various number of periods N and **a**: the same *b* = 5000 s/mm^2^ or **b**: the same gradient strength *G* = 300 mT/m.

This simulation analyses the angular signal dependence for DODE with various number of half periods *N* and substrates with different levels of microscopic diffusion anisotropy. Figure 4a plots the signal itself as well as the normalized signal with respect to the measurements with parallel gradients for DODE sequences with the same *b* = 5000 s/mm^2^. In this case, the amplitude modulation initially increases with *N*, then it decreases, a trend that can be explained by analyzing which components of the diffusion tensor spectrum D(ω) are sampled 54. In case of restricted diffusion, D(ω) increases with frequency, reaching the free diffusivity value for ω→∞. DODE sequences with *N* = 2 probe the smaller values of the diffusion spectrum at low frequencies, i.e., long diffusion times, and yield little signal attenuation for the given *b*‐value. DODE sequences with medium values of *N* start probing larger values of D(ω) and provide a higher signal attenuation as well as sensitivity to restriction. As the number of oscillations is further increased, DODE sequences probe even larger values of D(ω) which approach free diffusivity as ω→∞, and lose sensitivity to restriction.

Analyzing sequences with the same *b*‐values is important for understanding the effects of varying oscillation frequency, however, these sequences cannot be readily achieved in practice, as there is a physical constraint on the maximum gradient strength. Figure 4b presents the same dependence in a more practical situation, when the DODE sequences have the same gradient amplitude *G* = 300 mT/m. In this case, the diffusion weighting (*b*‐value) of different sequences varies over several orders of magnitude. The DODE sequences with a large number of oscillations yield little signal attenuation, while the DODE sequence with *N* = 2 attenuates the signal close to the noise floor in substrates with elongated pores. As illustrated in Figure 4b DODE sequences with an intermediate frequency yield the highest signal modulation amplitude. In all cases, the amplitude of the signal modulation decreases as the pores become more isotropic, as expected from previous studies on DDE sequences 16, 60.

### Study 2: Sensitivity of DODE and DDE Signal to Pore Size and Length

Depending on the restriction size, an optimal balance between gradient strength and oscillation frequency is required in order to maximize the sensitivity to microstructural features. The second part of this work analyses the sensitivity of DODE and DDE measurements to microstructural parameters, specifically pore diameter and length, which determine µA. We investigate the sensitivity for a variety of substrates as well as sequence parameters.

#### Sensitivity for a Wide Range of Substrates

**Figure 5:**
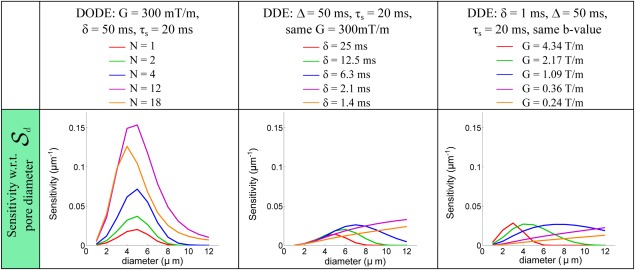
Sensitivity of DODE and DDE sequences with respect to pore diameters $S_{d}$ for substrates consisting of randomly oriented infinite cylinders. For each DODE sequence with number of periods *N*, the parameters of the DDE sequences were chosen as explained in section: DDE with finite pulses ( 2nd column) and DDE with short gradient pulses and the same *b*‐value (3rd column).

This simulation investigates the sensitivity of several DODE and DDE sequences in substrates consisting of either randomly oriented infinite or finite cylinders with a wide range of sizes and eccentricities. Figure 5 presents the dependence of sensitivity to pore diameter, $S_{d}$, for randomly oriented infinite cylinders. The sensitivity is calculated for DODE sequences with various *N* and the corresponding DDE sequences with the same gradient strength and finite duration as well as ideal DDE sequences with short gradient pulses and the same *b*‐value. To match *b*‐value, the gradient amplitude of the idealized short‐pulse DDE sequence must reach over 4T/m which is not practical even in most preclinical settings, but we include the results for theoretical comparison. DODE sequences show higher sensitivity than DDE for a range of pore diameters between 2 and 8 µm, as noted by the higher values of $S_{d}$. Conversely, DDE sequences, both with finite pulses as well as with short pulses, have higher sensitivity for larger pore diameters *d* > 8 µm. For these pore sizes, a longer diffusion time, which is achieved using DDE sequences, is necessary to better probe the pore boundaries. It is interesting to note that these results also hint at increased specificity, for example when N={12,18}, the signal is most sensitive to small diameters, and less sensitive to larger sizes. Nevertheless, for the range of pore diameters analyzed here, these sequences retain some sensitivity to larger pore diameters as well.

**Figure 6:**
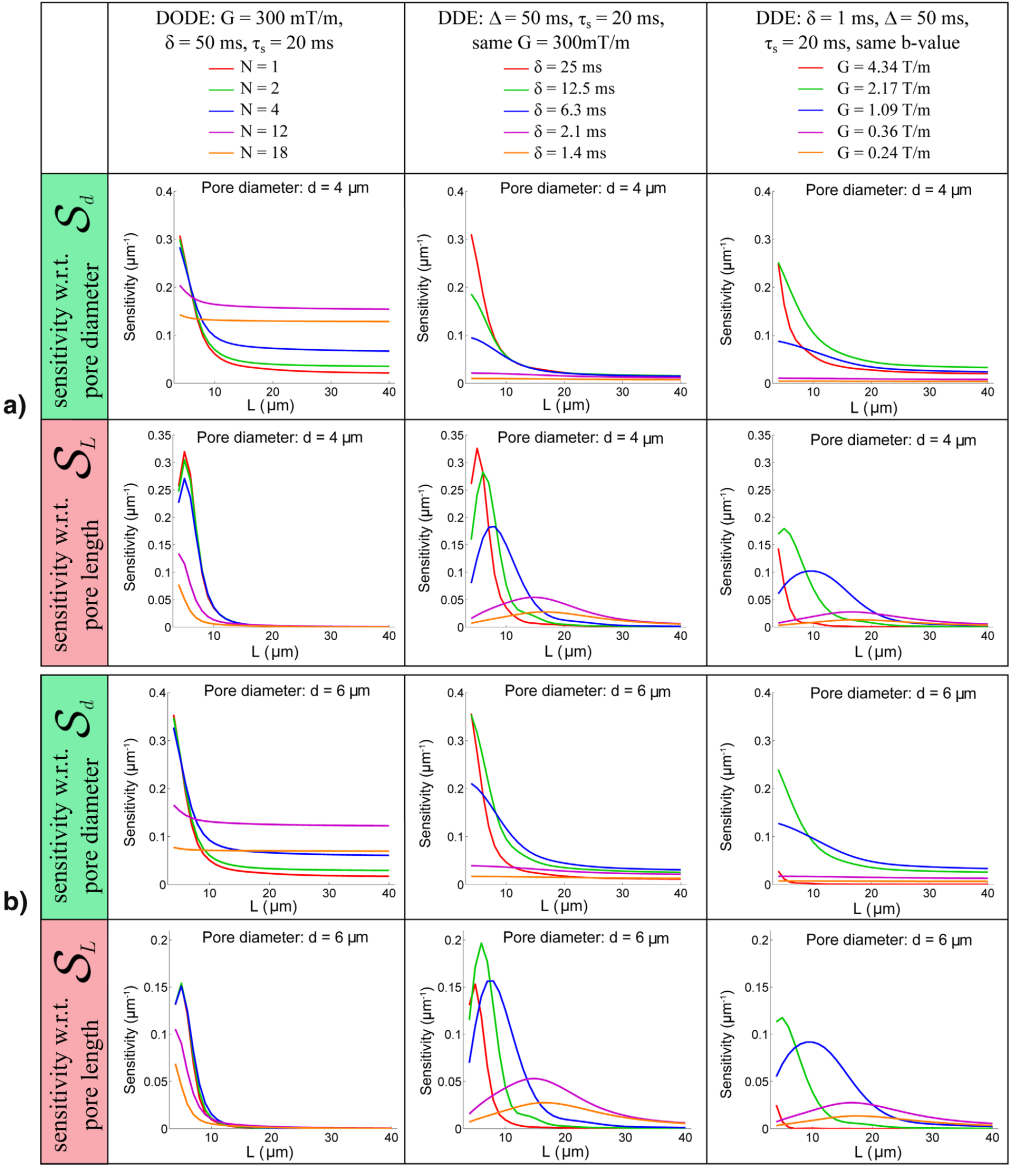
Sensitivity of DODE and DDE sequences with respect to pore size, $S_{d}$, and length, $S_{L}$, for randomly oriented finite cylinders with various lengths and two different diameters **a**: *d* = 4 µm and **b**: *d* = 6 µm. Different columns illustrate different sequences as explained in Figure 5.

Figure 6 illustrates the DODE and DDE sensitivities with respect to pore diameter and length, $S_{d}$ and $S_{L}$, in substrates consisting of randomly oriented finite cylinders. For elongated pores with *L* > 8 µm, DODE sequences with *N* = 12 provide the highest sensitivity to pore diameter, $S_{d}$, for both d={4,6} µm, which is consistent with the results in Figure 5. For less eccentric pores (*L* < 8 µm), DODE sequences have no net advantage. Nevertheless, the maximum sensitivity of DODE and DDE sequences with finite pulses is higher compared to values obtained from DDE sequences with short gradient pulses (SGP) (3rd column). When considering sensitivity to pore length $S_{L}$, DDE sequences with finite gradient duration provide the highest sensitivity in all substrates. The simulations show that ideal DDE sequences with short gradient pulses do not have any advantages in terms of sensitivity to microstructural features. For the diffusion times and gradient strengths considered in this study, the sensitivity $S_{L}$ of all sequences decreases almost to 0 for pores with *L* > 20 µm.

#### Sensitivity for a Wide Range of Sequence Parameters

To generalize the previous findings, a larger sampling of the sequence parameter space is required. This simulation analyses the sensitivity of DODE and DDE sequences over a large parameter space with practical values for several diffusion substrates.

**Figure 7:**
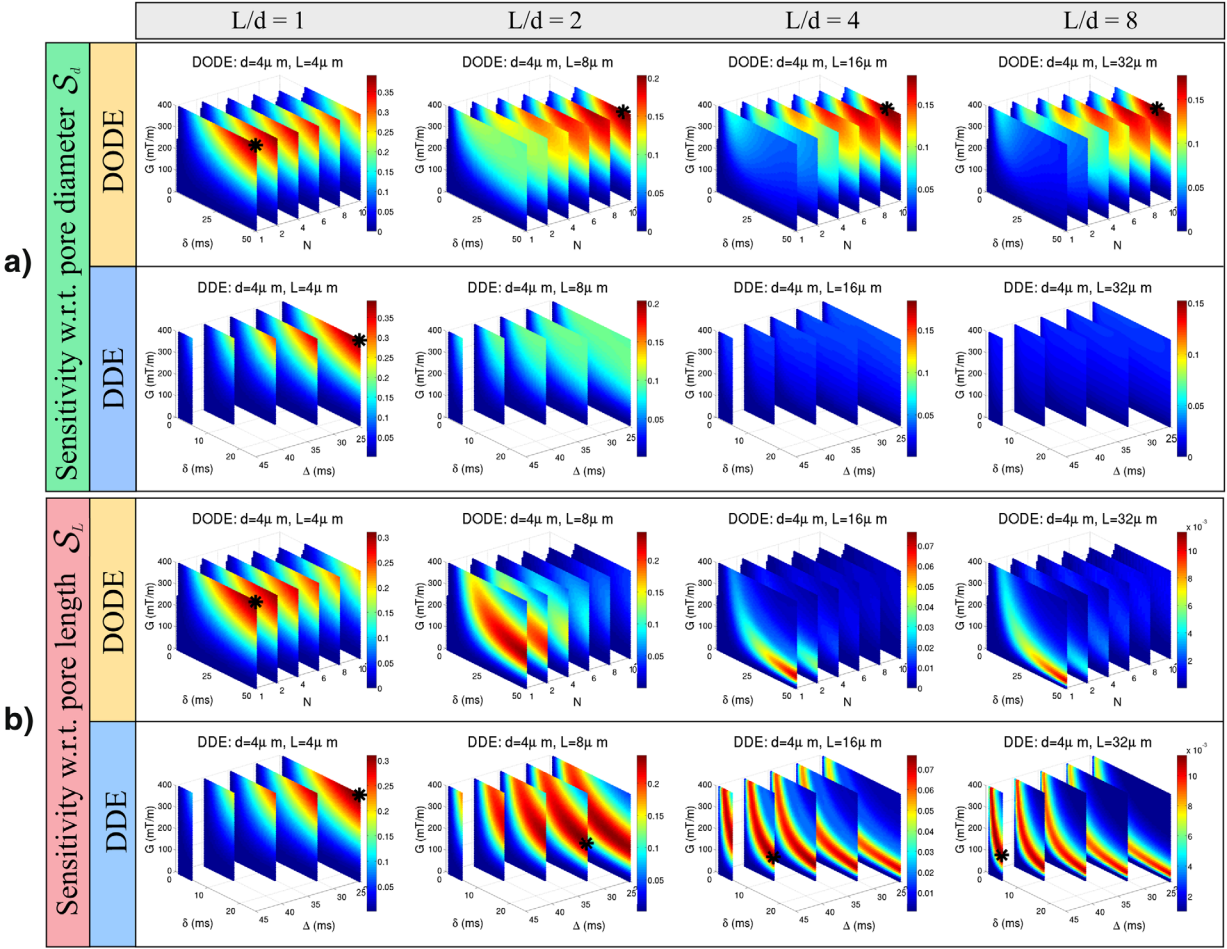
Sensitivity of DODE and DDE sequences with respect to **a**: pore diameter $S_{d}$ and **b**: pore length $S_{L}$ over a large parameter space. The diffusion substrates consist of finite cylinders with diameter *d* = 4 µm and different elongations ( L/d={1,2,4,8}). The maximum total duration is the same for DODE and DDE sequences and the effects of T2 relaxation are not taken into account. In each panel, the pore eccentricity increases from left to right. The signal sensitivity is color coded and the limit of the color bar depends on the substrate but is the same for DDE and DODE sequences. The asterisk depicts the most sensitive sequence for each substrate.

Figure 7 illustrates the sensitivities $S_{d}$ and $S_{L}$ for substrates with diameter *d* = 4 µm and various eccentricities, when the effects of T2 decay are not considered. The asterisk depicts the most sensitive sequences for each substrate. DODE sequences with the largest number of half periods considered in the parameter space (*N* = 10) are the most sensitive to pore diameter in elongated pores (*L* > 8 µm). If we allow higher frequencies, then, for the given limits of gradient strength and duration, the overall maximum sensitivity occurs for *N* = 14 and its values is higher by ∼20%. In substrates with isotropic pores of diameter *d* = 4 µm, DODE sequences with *N* = 1, which are equivalent to DDE sequences with Δ=δ have the highest sensitivity to pore size. When considering the sensitivity to pore length, DDE sequences with a low gradient strength and the longest pulse duration for the corresponding diffusion time are the best choice. The plots also illustrate that the diffusion time of the most sensitive DDE sequence increases with pore length, as larger length scales need to be probed.

**Figure 8:**
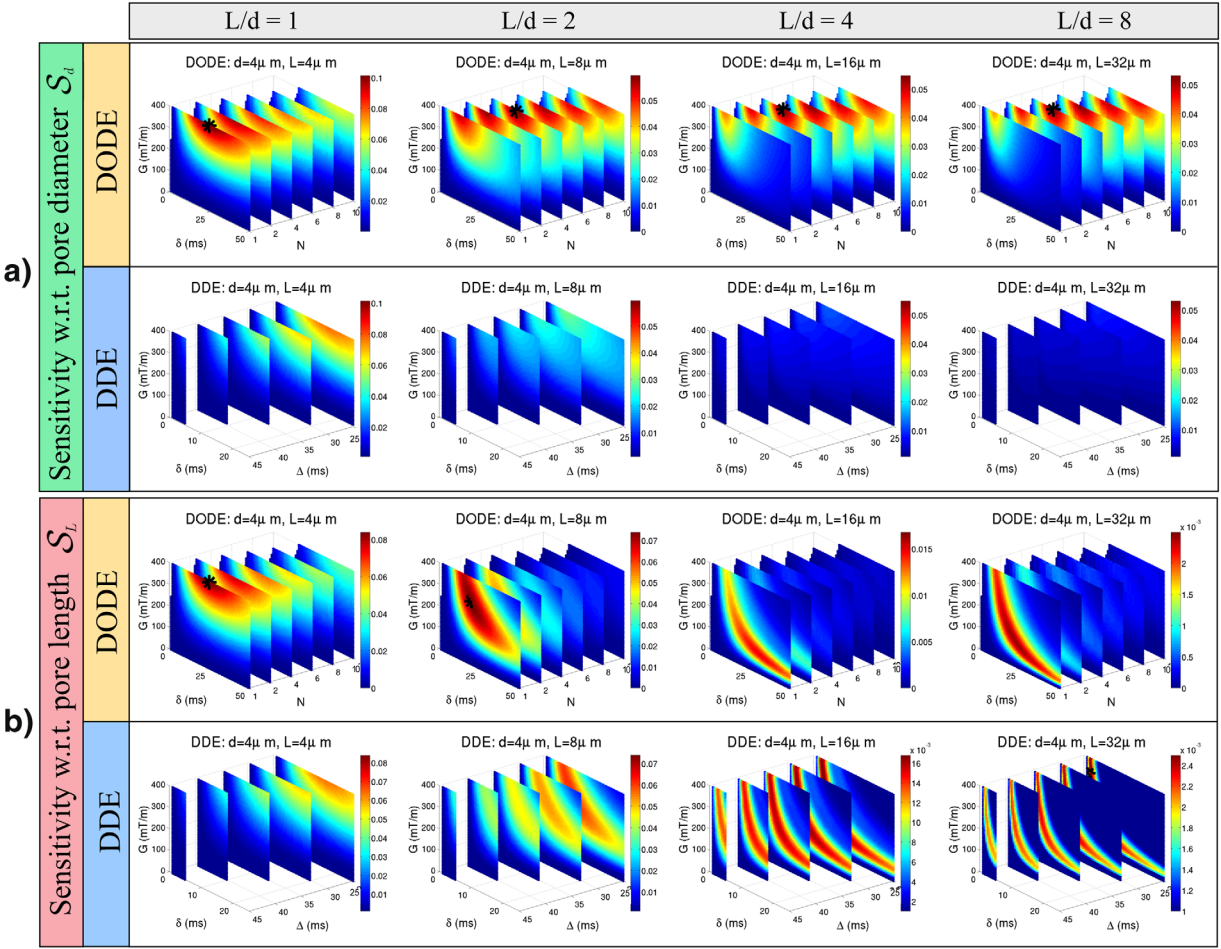
Sensitivity of DODE and DDE sequences with respect to **a**: pore diameter $S_{d}$ and **b**: pore length $S_{L}$ in the presence of T2 relaxation. All other parameters are the same as in Figure 7.

Figure 8 compares the sensitivity of DODE and DDE sequences, when the effects of T2 relaxation are taken into account with a relaxation constant T2 = 70 ms. In this case, DODE sequences with a lower number of oscillations (*N* = 4) and shorter gradient duration, compared to the results in Figure 7a, show the highest sensitivity to pore diameter in elongated pores. The optimal sensitivity to pore length in elongated pores (*L* > 16 µm) is still achieved by DDE sequences with Δ>δ, while for less elongated pores DDE sequences with Δ=δ are preferred. Nevertheless, the optimal parameter values are different. When T2 decay is considered, the preferred DDE sequences have larger gradient strength and shorter pulse duration and diffusion time compared to the results in Figure 7b.

Overall, the results show that a combination of DODE and DDE sequences provides complementary sensitivity to different microstructural features such as pore diameter and length.

### Study 3: Specificity to Microscopic Anisotropy

**Figure 9:**
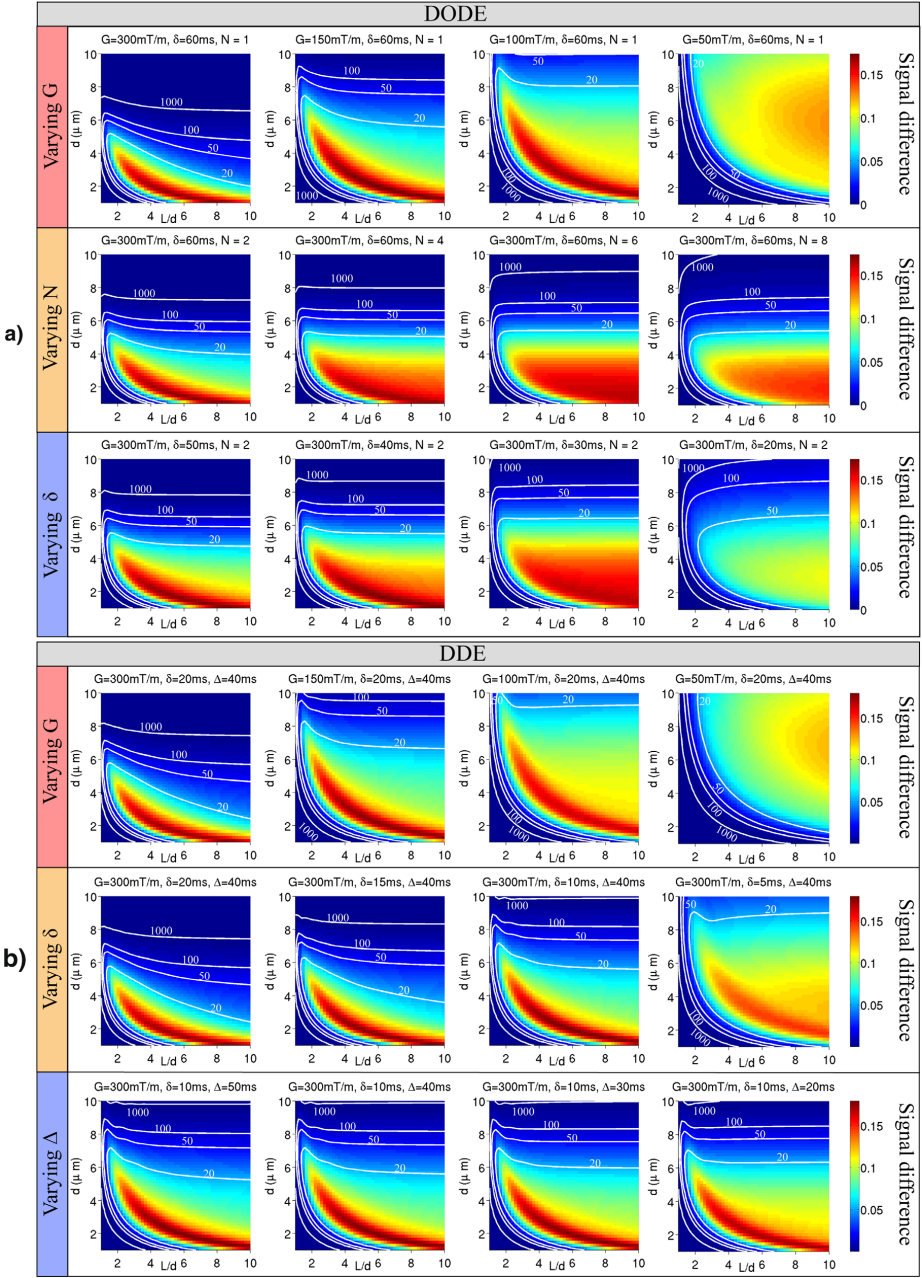
Difference between parallel and perpendicular measurements of **a**: DODE and **b**: DDE sequences as a function of pore size and eccentricity. In each row, a different sequence parameter is varied, while all the other parameters are constant. The white contours indicate the limit where the difference is equal to the standard deviation of noise for SNR={20,50,100,1000}.

The last simulation investigates how the signal difference between DDE/DODE measurements with parallel and perpendicular gradients depends on the sequence parameters in various substrates. Figure 9 presents the signal difference as a function of pore size and eccentricity. Different rows in panels (a) and (b) have sequences with different varying parameters. Sequences with large gradient strength are more sensitive to smaller pore sizes, and decreasing G shifts sensitivity to larger and more elongated pores for both DODE and DDE sequences. For DODE with varying *N*, a slightly different pattern is observed, with enhanced sensitivity sensitivity to pore diameter for elongated pores, i.e., there is a stronger color gradient in vertical direction for the entire range of eccentricities. Decreasing the gradient duration has an overall effect of reducing the sensitivity due to a decrease in diffusion weighting. For DDE sequences, decreasing *δ* while having a long diffusion time has a similar effect to increasing *N* for DODE, nevertheless, the effect is less pronounced. For DDE sequences, increasing diffusion time improves sensitivity to pore elongation, which can be seen as a sharper gradient in panel (b) bottom row. All in all, this simulation shows that DODE and DDE sequences with different parameters are required in order to estimate different microstructural properties. Thus, for substrates with unknown microstructural features or in areas with a superposition of cellular structures, measurements with a range of different parameters are needed. A careful choice of sequence parameters can also be used to enhance the signal acquired from a certain tissue configuration, while suppressing the signal from different ones.

## DISCUSSION

This article introduces a novel class of diffusion sequences, namely DODE, and explores the sensitivity of DODE and DDE sequences to microstructural features in substrates which only exhibit microscopic diffusion anisotropy. DODE sequences increase the sensitivity to pore diameter in the range of 2–8 µm, while DDE sequences are more sensitive to pore length. Furthermore, the sequence parameters can be adjusted to enhance the specificity to a particular range of substrates, which can be useful in experiment design.

In the first study, we compared the DODE and DDE signal for sequences with various separation times and oscillation frequencies. As the angle *ψ* between the two gradient waveforms varies, the DODE measurements also exhibit the characteristic amplitude modulation, which is well‐known for DDE sequences. However, as the number of oscillations in the DODE sequence increases, the effect of varying separation time becomes less pronounced compared to DDE, which can be better understood in the frequency domain. For instance, as illustrated in Figure 3, changing the orientation of the second gradient from parallel to antiparallel splits in the main peak of the power spectrum for DODE and DDE sequences with zero separation time. For DDE sequences, this peak is around zero‐frequency, and the diffusivity values sampled in the two cases are very different, while for DODE sequences the difference becomes smaller as the frequency increases.

The effect of different oscillation frequencies was investigated for DODE measurements with the same *b*‐value or the same gradient strength. For sequences with the same *b*‐value, the peak amplitude in the power modulation spectrum is the same for different frequencies, however, as *N* increases the gradients probe the higher diffusivity values corresponding to less restricted diffusion and the signal attenuation increases. For large *N*, the amplitude of the signal modulation as a function of *ψ* decreases, reflecting smaller values of microscopic anisotropy on the respective time‐scales. When DODE sequences have the same gradient strength, their diffusion weighting (*b*‐value) varies over several orders of magnitude, and a fine balance between signal attenuation and sensitivity to restriction needs to be achieved.

The next set of simulations analyzed the sensitivity of DODE and DDE sequences to pore diameter and length, with the aim of identifying regimes where each sequence is beneficial. The first analysis is focused on sequences with several parameter combinations and a large variety of diffusion substrates. In randomly oriented infinite cylinders, we found that DODE sequences improve the sensitivity to pore diameter for a range of values between 2 and 8 µm. This is consistent with Drobnjak et al.'s findings, showing higher sensitivity for OGSE sequences in cylindrical pores with orientation dispersion 28. The advantage of DODE arises from less attenuation due to diffusion along the long axis of the pore, while preserving sensitivity to restriction. When considering substrates of finite cylinders, DODE sequences improve sensitivity to pore diameter in elongated pores, while DDE acquisitions have higher sensitivity to pore length. Furthermore, we found that ideal DDE sequences with short gradient duration and the same *b*‐value do not necessarily have an advantage with respect to sensitivity to microstructural features.

The subsequent simulation examined the sensitivity of sequences over a wide range of practical sequence parameters in several substrates. This analysis further demonstrates that DODE sequences show higher sensitivity to pore diameter in elongated pores, while DDE sequences have larger sensitivity to pore length. This trend was observed both when T2 decay was neglected or considered in the sensitivity measure, however, the optimal parameters look different in the two cases. When the effects of T2 are neglected, the maximum sensitivity for DODE sequences occurs at long pulse durations and the largest *N* considered in the parameter space. When *N* is further increased, the sensitivity eventually decreases, as the diffusion time becomes too short to probe restricting boundaries. For sensitivity to pore length, the optimal DDE measurements have a low gradient strength and the longest pulse duration which can be achieved for the preferred diffusion time. Moreover, longer diffusion time allows larger length scales to be probed more accurately. When T2 effects are considered, the optimal DODE sequences have shorter pulse duration and a lower number of oscillations, and the optimal DDE sequences have shorter duration and higher gradient strength compared to the case of infinite T2. DDE sequences with Δ=δ have the highest sensitivity to small, isotropic pores, as they maximize the amount of diffusion weighting for a given duration. Their advantage is perhaps somewhat surprising, as many DDE‐based studies to date have opted using DDE sequences resembling as much as possible to the ideal SGP limits 10, 11, 14, 17, although others used longer gradient durations due to gradient amplitude constraints 5, 20, 21, 62. These results suggest that diffusion protocols which combine DODE and DDE measurements would be sensitive to a wide range of configurations and pore‐sizes. Combining oscillating and pulsed gradients is also beneficial for estimating cell size, volume fraction and intrinsic diffusivity, as has been previously illustrated for restricted diffusion inside spherical cells 63. Moreover, when there is prior knowledge of the substrates, the sequences can be optimized to improve the sensitivity to a particular configuration.

The last study points to a potential specificity of DDE/DODE sequences towards different µA. A given pixel within brain tissues, particularly gray matter, will reflect a superposition of several very different environments for water diffusion. For instance, large and approximately spherical cell bodies may co‐exist with randomly oriented neurites 64. The results presented in Figure 9 show that by changing the sequence parameters we can manipulate which substrates the sequence would be most sensitive to, based on the respective signal differences between parallel and perpendicular gradients. Although the plots in Figure 9 do not show very localized maxima, further investigations optimizing these signal differences in DDE/DODE toward specific microstructures could be beneficial. This approach would be especially useful for estimating model‐free metrics based on the signal difference.

In this work, we analyzed gradient waveforms with a high slew rate suitable for preclinical scanners (SR = 1000 T/m/s) and a diffusion substrate featuring intracellular space only, nevertheless, the conclusions are similar when more realistic sequences and substrates are used to compare the sensitivity of DODE and DDE sequences. In the Supporting Figures S1 and S2, we present the sensitivity analysis for waveforms with SR = 200 T/m/s when the effects of T2 relaxation are taken into account or not. As recent studies have shown that extracellular space also exhibits a time dependent diffusivity 66, 67, in Figure 3 we show the sensitivity results generated using Monte Carlo simulations 68 which provide a more realistic model for the extracellular space.

The analyzed DODE sequences have cosine‐like gradient waveforms, which could be challenging to use on clinical systems with low gradient strength, due to their limited diffusion weighting. We have also performed all the simulations for DODE sequences with sine waveforms, which yield higher diffusion weighting at the same frequency. The results show the same advantages of sine DODE over DDE. However, in simulations similar to the ones in Figures 7 and 8, sine waveforms yield a smaller maximum sensitivity ( ∼20%) compared to cosine DODE, due to their zero‐frequency peak in the power modulation spectrum 54.

In this work, we focused on macroscopically isotropic substrates. Nevertheless, we expect all conclusions from this study to apply equally well with rotationally invariant acquisitions, e.g., following the gradient direction schemes presented for DDE sequences in 12, 13, 14. This allows the estimation of microscopic anisotropy in a wide variety of tissues, which might also exhibit macroscopic anisotropy.

This analysis is concentrated on comparing DODE and DDE type acquisitions in the context of angular experiments as well as their sensitivity to microstructural parameters. A comparison with other diffusion sequences that have been recently presented in the literature for estimating microscopic anisotropy 6, 22, 23, 24 is outside the scope of this work and will be considered in future research.

DODE sequences can also be readily set up on clinical and preclinical scanners. Oscillating gradients have been previously implemented for OGSE studies, and converting them to a DODE type acquisition requires changing just the orientation of the second lobe. On clinical scanners, constraints on gradient slew rate due to hardware as well as peripheral nerve stimulation and cardiac stimulation, limit the frequency of oscillating gradients, nevertheless, the sequences which show the maximum sensitivity to pore size have a low number of periods. Future work aims to implement and validate DODE sequences, both for estimating pore size and eccentricity in a model‐based framework, e.g., 65, as well as for computing indices of microscopic anisotropy.

## Supporting information


**Fig. S1.** Sensitivity of DODE and DDE sequences with slew rate *SR* = 200T/m/s with respect to a) pore diameter $S_{d}$ and b) pore length $D_{L}$, when considering the effects of T2 relaxation are not taken into account. The rest of the parameters are the same as in Figure 7.
**Fig. S2.** Sensitivity of DODE and DDE sequences with slew rate SR = 200T/m/s with respect to a) pore diameter $S_{d}$ and b) pore length $D_{L}$, when considering the effects of T2 relaxation. The rest of the parameters are the same as in Figure 7.
**Fig. S3.** Sensitivity of DODE and DDE sequences with respect to pore diameter with or without considering T2 relaxation, when the gradient a) is perpendicular to the cylinder axis or b) deviates from orthogonality by a 5° angle.
