# Supplementary material for: Double oscillating diffusion encoding and sensitivity to microscopic anisotropy
Source: Magn Reson Med. 2016 Aug 31;78(2):550–64. doi: 10.1002/mrm.26393 (PMC5516160; doi:10.1002/mrm.26393)
Supplement: Supplementary file 1 — Fig. S1. Sensitivity of DODE and DDE sequences with slew rate SR = 200T/m/s with respect to a) pore diameter Sd and b) pore length DL, when considering the effects of T2 relaxation are not taken into account. The rest of the parameters are the same as in Figure 7. Fig. S2. Sensitivity of DODE and DDE sequences with slew rate SR = 200T/m/s with respect to a) pore diameter Sd and b) pore length DL, when considering the effects of T2 relaxation. The rest of the parameters are the same as in Figure 7. Fig. S3. Sensitivity of DODE and DDE sequences with respect to pore diameter with or without considering T2 relaxation, when the gradient a) is perpendicular to the cylinder axis or b) deviates from orthogonality by a 5° angle. [file MRM-78-550-s001.pdf]

Supporting material

DODE and DDE sensitivity for sequences with a smaller gradient slew rate

This analysis presents the sensitivity of DODE and DDE sequences to pore size and length for a wide range of sequence parameters, when the slew rate of the gradient is smaller, i.e. $SR = 200\text{T/m/s}$, a value that can be achieved on the high-performance Connectome scanner. The rest o the parameters are the same as in Study 2: The diffusion substrates consist of randomly oriented finite cylinders with $d = 4\mu\text{m}$ and $L/d = \{1, 2, 4, 8\}$ and the range of sequence parameters are the following: for DODE sequences are we have $G = [0, 400]$ mT/m, $\delta_{DODE} = [0, 50]$ ms and $N = \{1, 2, 3, 4, 5, 6\}$, and for the DDE sequences we have $G = [0, 400]$ mT/m, five different diffusion times $\Delta = \{25, 30, 35, 40, 45\}$ ms and gradient duration s.t. $\delta + \Delta < 50$ ms. The separation time is fixed to 20 ms for all sequences. As in Study 2, we investigate the sensitivity with and without the effects of T2 relaxation.

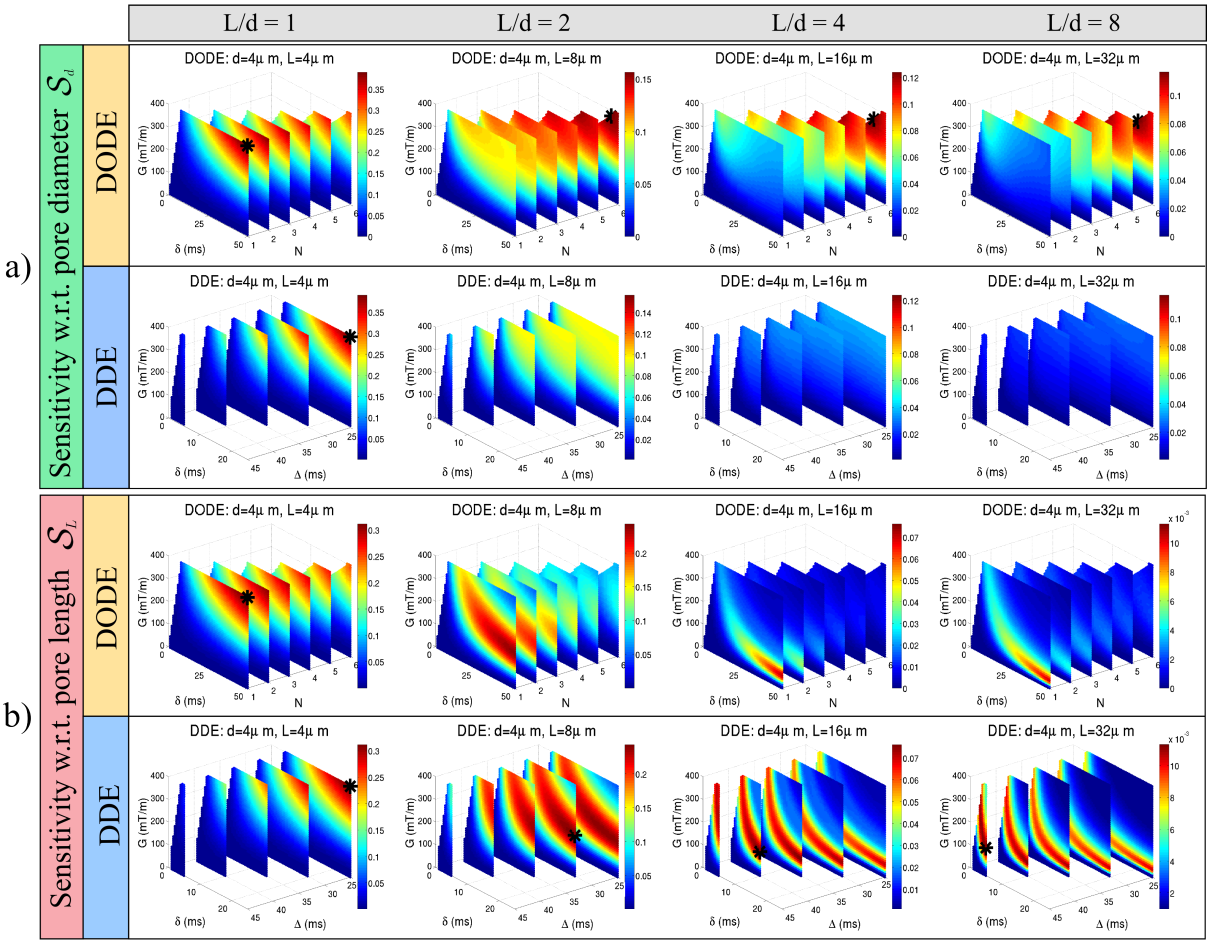

Figure S1: Sensitivity of DODE and DDE sequences with slew rate $SR = 200\text{T/m/s}$ with respect to a) pore diameter $\mathcal{S}_d$ and b) pore length $\mathcal{D}_L$, when considering the effects of T2 relaxation are not taken into account. The rest of the parameters are the same as in Figure 7.

Figures S1 and S2 plots the sensitivity of DODE and DDE sequences with a much smaller slew rate $SR = 200\text{T/m/s}$. Although, DODE sequences with high frequencies cannot be achieved due to slew rate limitations, we see the same pattern as in Figures 7 and 8 from the main text: DODE sequences present more sensitivity to pore diameter, while DDE sequences have more sensitivity to pore length.

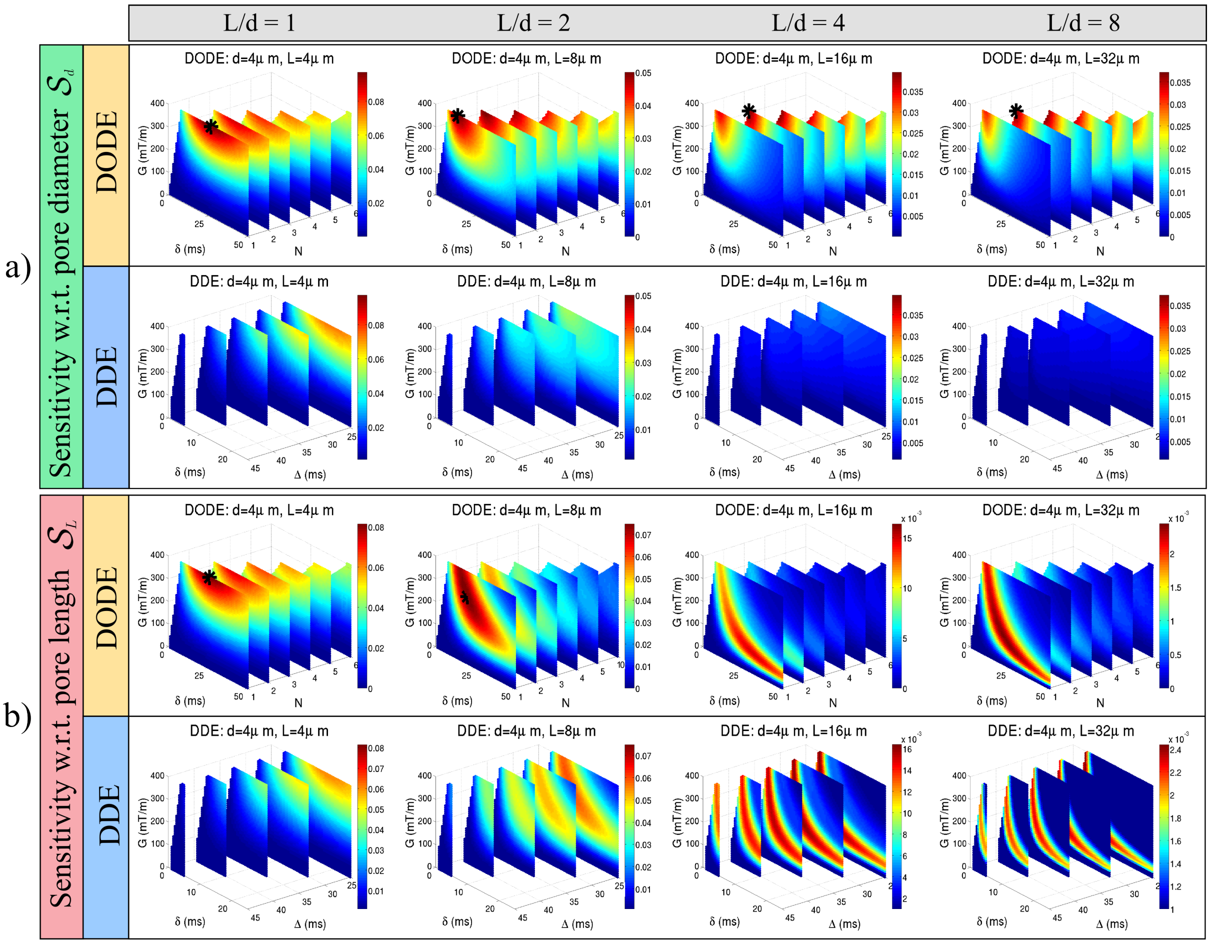

Figure S2: Sensitivity of DODE and DDE sequences with slew rate $SR = 200$T/m/s with respect to a) pore diameter $\mathcal{S}_d$ and b) pore length $\mathcal{D}_L$, when considering the effects of T2 relaxation. The rest of the parameters are the same as in Figure 7.

DODE and DDE sensitivity to pore diameter in the presence of extracellular diffusion

As recent research has shown, diffusivity in the extracellular space is also time-dependent [66, 67], therefore, in this simulation we test the effects of diffusion in the extracellular space on the sequence sensitivity to pore diameter. Thus, we use the Monte Carlo diffusion simulator in Camino [68] to synthesize diffusion data both in the intra- and extracellular environments. As packing randomly oriented elongated pores with a high volume fraction is challenging [65], for this proof of concept we use a substrate consisting of randomly placed parallel cylinders with diameter $d = 4\mu$m and a volume fraction of 61%. We investigate the sensitivity of DODE and DDE sequences with parallel gradients in two cases, when the gradient orientation is either perpendicular or not perfectly perpendicular (5° deviation) to the cylinder axis. The sequence parameters are the same as for the simulations presented in Figures 7 and 8.

The plots in Figure S3 show the DODE and DDE sensitivity to pore diameter when diffusion in the extracellular space is accounted for. The maps appear slightly noisy due to discretization in numerical Monte Carlo simulations as opposed to the semi-analytical approach used in the main text, however the overall patterns are very similar. Although modelling diffusion in the extracellular space may provide a more accurate signal description [66], these results show that for the substrates considered here, a model of intracellular diffusion is good enough to study the contrast given by different sequences.

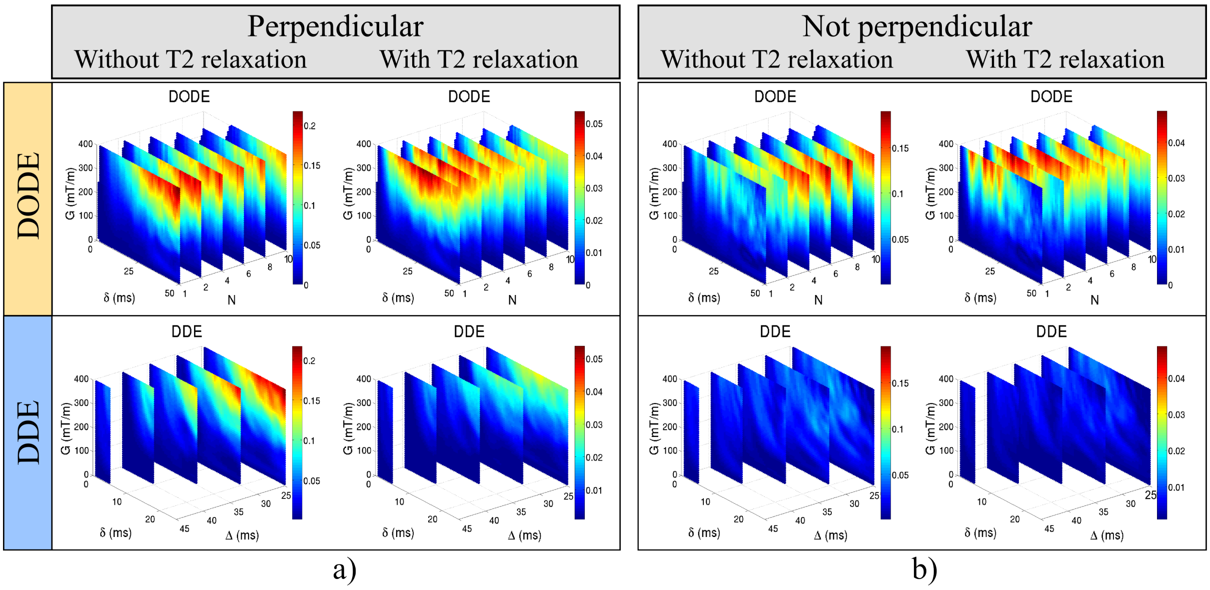

Figure S3: Sensitivity of DODE and DDE sequences with respect to pore diameter with or without considering T2 relaxation, when the gradient a) is perpendicular to the cylinder axis or b) deviates from orthogonality by a 5° angle.
